# Application value of ^18^F-FDG PET/CT in soft tissue metastasis of intrahepatic cholangiocarcinoma: a case report and literature review

**DOI:** 10.3389/fonc.2024.1474105

**Published:** 2025-01-16

**Authors:** Siwen Liu, Xiaohui Sun, Yu Liu, Ning Shi, Xiaoli Zhang, Yuechao Yu

**Affiliations:** Department of Nuclear Medicine, The Second Affiliated Hospital of Xuzhou Medical University, Xuzhou, Jiangsu, China

**Keywords:** PET/CT, intrahepatic cholangiocarcinoma (ICC), soft tissue, metastasis, ^18^F-FDG

## Abstract

Intrahepatic cholangiocarcinoma (ICC)originates from the epithelial cells of the intrahepatic bile ducts, with insidious onset and strong invasiveness, and most of the cases are found in the advanced stage, with extremely poor prognosis. In advanced stages, distant metastases to the lungs, bones, and brain are common, but distant soft tissue (subcutaneous and skeletal muscle) and breast metastases are rare, and simultaneous metastases to all three rare sites had not been reported. We report a 69-year-old woman with right upper abdominal pain who underwent a plain and enhanced CT scan of the upper abdomen, which revealed an intrahepatic space-occupying lesion, as well as subcutaneous and peritoneal nodules in the abdomen. To further evaluate the presence of other metastases, an ^18^F-FDG PET/CT scan was performed, which showed abnormal FDG uptake in the liver, peritoneum, left upper femur, right breast, subcutaneous tissues of the thoracic and abdominal regions, and skeletal muscle, while the corresponding CT densities of part of the skeletal muscle and the left upper femur did not show any significant abnormality. Pathologic confirmation of ICC with multiple metastases was obtained by puncture biopsy of the liver and subcutaneous nodes. This case demonstrates the advantages of ^18^F-FDG PET/CT in comprehensively evaluating systemic metastasis of ICC and detecting occult metastases, which is of great significance in its clinical diagnosis and staging.

## 1 Introduction

ICC is a rare malignancy that originates from the epithelial cells of the intrahepatic bile ducts. Its onset is insidious, early clinical symptoms are not obvious, and the disease progresses rapidly, many patients are already in the advanced stage when diagnosed, and the prognosis is often very poor (1). ICC can metastasize in the lymphatic chain, including the hepatoduodenal ligament, and it often invades neighboring organs or metastasizes to other internal organs, such as lungs, bones, adrenal glands, and brain. In contrast, subcutaneous soft tissue, skeletal muscle, and breast metastases are rare (2). In addition, distant soft tissue metastatic tumors rarely exhibit specific symptoms, making them difficult to recognize on routine examination. As the application of PET-CT in tumor diagnosis becomes more and more widespread, its value for qualitative diagnosis and staging of tumors has been affirmed (3, 4). The fusion of CT’s precise anatomical structure imaging and PET’s sensitive metabolic imaging has obvious advantages in the definitive diagnosis of metastatic foci in areas that are not easily metastasized, such as skeletal muscles. Currently, distant metastases of cholangiocarcinoma to skeletal muscle, subcutaneous soft tissues, and breast have only been reported in sporadic case reports or series. In contrast to previous reports of skeletal muscle metastases from ICC, the present patient had already developed distant soft tissue metastases before common sites of metastasis, such as the liver and lymph nodes, had occurred (5, 6). Very few reports of subcutaneous soft tissue metastases from ICC have been published. Most of these were derived from direct tumor seeding by percutaneous procedures. The scalp is the most common site of cutaneous metastasis of cholangiocarcinoma. In contrast, this report reports distant subcutaneous soft tissue metastasis of ICC limited to the chest and abdomen (7, 8). The articles already published did not reveal any reports of breast metastases originating from ICC. Subcutaneous soft tissue, skeletal muscle, and breast metastases are rare in ICC.ICC with metastases to skeletal muscle, subcutaneous soft tissue, and breast at the same time had not been reported. Here we report the first case of ICC with subcutaneous soft tissue, skeletal muscle, and breast metastases. The purpose of this case is to provide clinicians and radiologists with a more comprehensive understanding of this metastatic tumor so that they can consider the possibility of this metastatic pattern when encountering similar cases in the future.

## 2 Case presentation

**Figure 1:**
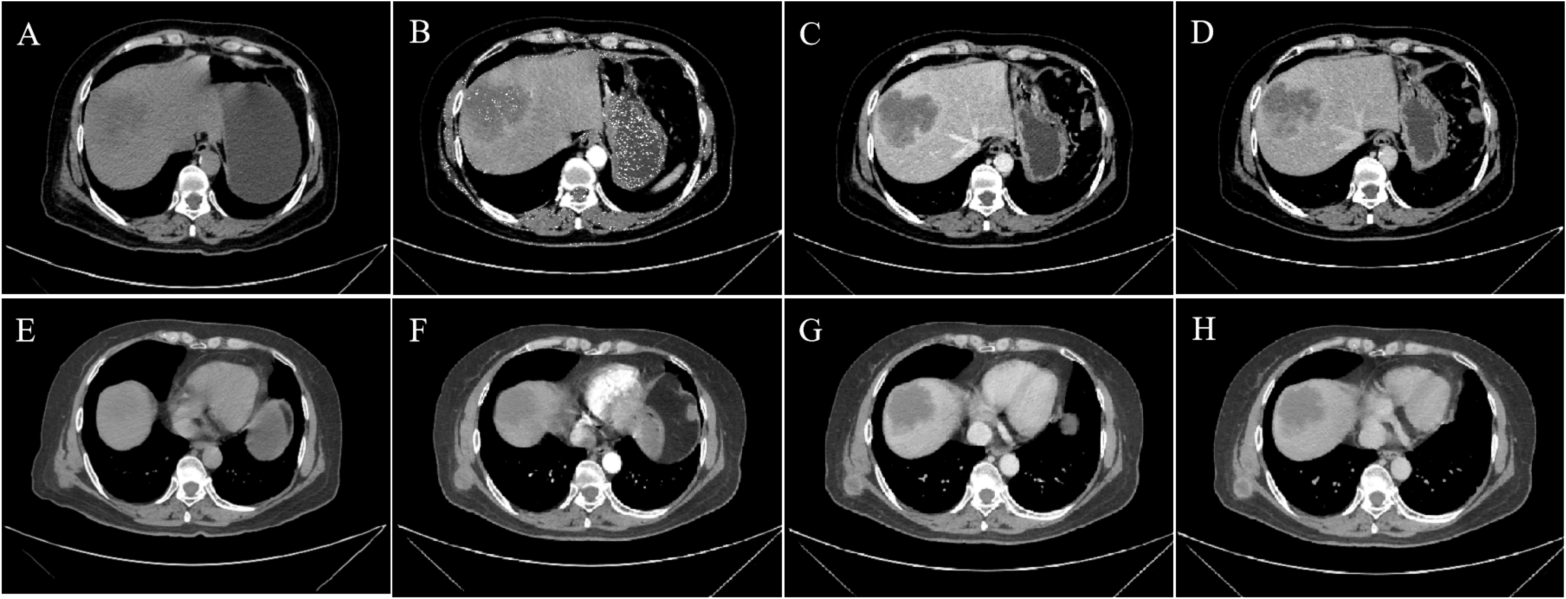
CT and Contrast-enhanced CT imaging images. **(A, E)** Non-contrast-enhanced CT, **(B, F)** enhanced CT-arterial phase, **(C, G)** enhanced CT-venous phase, and **(D, H)** enhanced CT-delayed phase. **(A)** The non-contrast-enhanced CT scan of the abdomen showed an irregular mass in the right lobe of the liver with unclear borders and uneven density. **(B–D)** Enhanced CT scan showed heterogeneous delayed enhancement at the margin of the lesion in the right lobe of the liver,with areas of nonenhanced necrosis in the central area of the lesion. **(E)** CT scan revealed a subcutaneous nodule on the right back. (**F–H)** Enhanced CT showed circular enhancement.

A 69-year-old woman presented with right upper abdominal pain in February 2024, which worsened after eating greasy food and was accompanied by lower back pain, and was treated with oral anti-inflammatory tablets and cephalosporin antibiotics, with unsatisfactory results. In March 2024, he went to the local hospital. Physical examination on admission showed localized pressure and pain in the right upper abdomen, and a hard nodule of about 2cm×2cm in size was seen on the right posterior chest wall, which was movable and accompanied by pressure and pain. The patient denied tumor and surgical history and family history of malignancy. Tumor markers showed elevated CA125 (43.6 U/ml, reference range: 0-35 U/ml), elevated CA199 (>1000.0 U/ml, reference range: 0-27 U/ml), and elevated SF (582.7 ng/ml, reference range: 30-400 ng/ml), while other laboratory tests did not show any significant abnormalities. CT scan of the upper abdomen showed an irregular mass in the right lobe of the liver, measuring about 7.8cm×6.4cm×6.3cm, with unclear borders and uneven densities (**Figure 1A**), and multiple nodules could be seen in the subcutaneous area of the chest and abdomen (**Figure 1E**) as well as in the peritoneum. Enhanced CT scan showed heterogeneous delayed enhancement at the margin of the lesion in the right lobe of the liver, with areas of nonenhanced necrosis in the central area of the lesion (**Figures 1B–D**), and partial circular enhancement in the subcutaneous thoracic dorsal area (**Figures 1F–H**) and peritoneal nodes. Combining the clinical and imaging features, it was suspected that there was an intrahepatic malignant tumor with multiple metastases.

**Figure 2:**
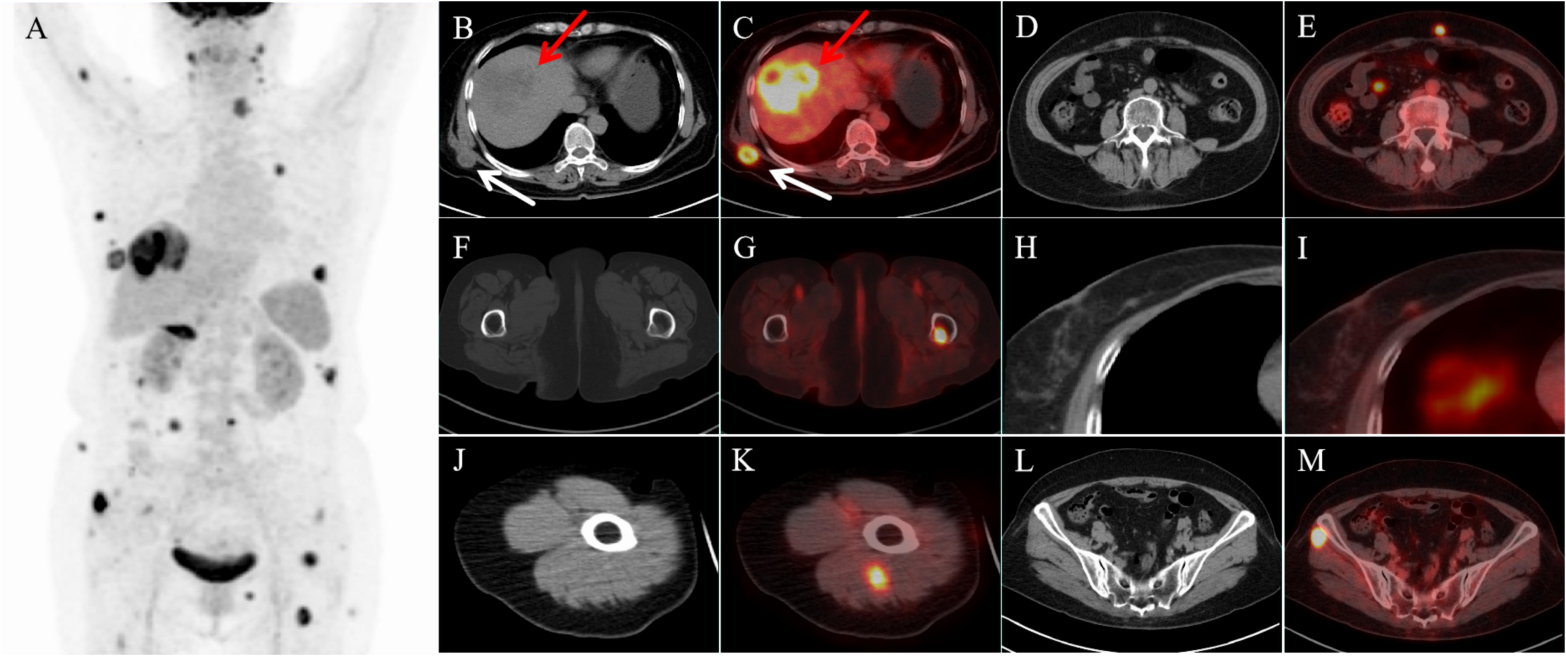
18F-FDG PET/CT images. **(A)** Maximum intensity projection (MIP) image demonstrated multiple lesions with abnormally elevated FDG uptake. Axial CT (**B**, red arrow) and transaxial fused PET-CT images (**C**, red arrow) showed a strong increased 18F-FDG uptake in the right lobe lesion of the liver. Transaxial fusion PET-CT images showed multiple hypermetabolic lesions in the abdominal subcutaneous tissue (**C**, white arrow), peritoneum **(E)**, femur **(G)**, right breast **(I)**, and some skeletal muscles **(K, M)**. Corresponding CTs of part of the skeletal muscles **(J, L)** and the left upper femur **(F)** did not reveal significant abnormal density shadows.

To fully evaluate the presence of other metastases, ^18^F-FDGPET/CT was performed in our department. Many lesions with abnormal FDG uptake were observed on the whole-body maximum intensity projection image (**Figure 2A**).In addition to foci of increased FDG metabolism in the right lobe of the liver (SUVmax15.3; **Figures 2B, C**, red arrow), thoracic and abdominal subcutis (SUVmax7.6; **Figures 2B, C**, white arrow), and peritoneum (SUVmax7.9; **Figures 2D, E**), the PET-CT axial fusion image also demonstrated increased metabolism in the left upper femur (SUVmax10.3; **Figures 2F, G**), right breast (SUVmax1.5; **Figures 2H, I**), right upper arm (SUVmax8.9; **Figures 2J, K**), and bilateral gluteal muscles (SUVmax15.8; **Figures 2L, M**) with multiple FDG hypermetabolic nodules, and the corresponding CTs of part of the skeletal muscles (**Figures 2J–M**) and the left upper femur (**Figures 2F, G**) did not show apparent abnormal density shadows.

**Figure 3:**
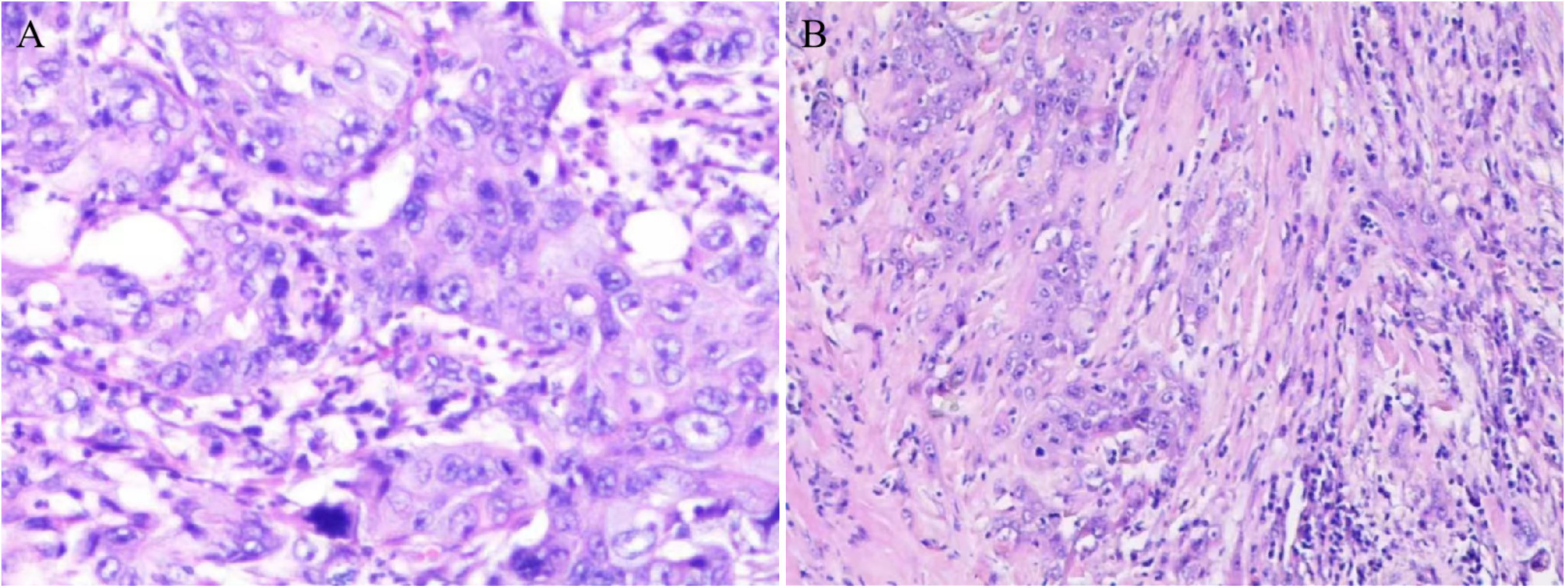
Pathologic picture. **(A)** Hepatic puncture biopsy pathology revealed hypo-differentiated cholangiocarcinoma. **(B)** Pathology of the dorsal mass showed metastatic adenocarcinoma.

Pathologic images of the patient’s hepatic puncture biopsy (**Figure 3A**, HE staining; The original magnification of 10×20) showed Hypo-differentiated cholangiocarcinoma. Immunohistochemistry: CAM5.2(+), CK (+), VI (+), LCA (-), CD5(-), CD20(-), Ki67(20%+), CK7(+), CK19(+), Muc-1(+), CK20(+), Villin (mostly weakly +), Muc-5(-), S1OOP (foci+), CRP (-). An ultrasound-guided puncture biopsy of the dorsal mass was performed, and the pathologic images (**Figure 3B**, HE staining; The original magnification was 10 × 10) suggest metastatic adenocarcinoma. Immunohistochemistry: CK pan (+), TTF1 (8G7G3/1) (-), Napsin A (-), GATA3 (-), CK7 (+), CK20 (individual +). A final diagnosis was ICC involving the peritoneum, left femur, subcutaneous tissue, skeletal muscle, and breast. The patient had undergone 7 cycles of gemcitabine plus cisplatin chemotherapy and is now on maintenance therapy with lapatinib. As of October 28, 2024, the patient’s vital signs were stable. Our team will continue to closely monitor this patient’s progress.

## 3 Discussion

ICC is a rare primary liver tumor with a poor prognosis. It is usually highly metastatic and invasive, so it has a higher probability of recurrence and metastasis. With the progression of the disease, advanced ICC tends to preferentially metastasize to the liver and extrahepatic organs, including the lungs, bones, brain, and lymph nodes. In contrast, metastasis to skeletal muscle, subcutaneous soft tissues, and breasts is very rare (9). Here, we report a rare case of ICC with subcutaneous, skeletal muscle, and breast metastases by PET/CT.

Early-stage ICC is challenging to diagnose because the clinical features of early-stage ICC are more subtle (most early-stage patients are asymptomatic), leading to an advanced stage by the time the patient is diagnosed.CA199 and CEA are the most commonly used serum markers for the diagnosis of ICC. It is worth noting that CA199 levels >1000 U/ml are associated with the presence of occult metastatic disease, but clinical and laboratory results are of limited diagnostic utility with low sensitivity and specificity (10, 11). Therefore, imaging plays an important role in the early detection and diagnosis of this tumor, and CT and MRI are currently the main imaging methods for the diagnosis of ICC. Dynamic enhancement CT of ICC is typically characterized by specific signs, including peripheral enhancement in the arterial phase of enhancement, peripheral contouring in the portal venous phase, and delayed central enhancement in the delayed phase. Retraction of the hepatic envelope, and dilatation of the adjacent bile ducts, sub-nodules, and/or satellite nodules are complementary signs in the diagnosis of ICC. The imaging characteristics of MRI are high signal in T2-weighted images and low signal in T1-weighted images; MRI enhancement scans may show peri-tumor enhancement in the arterial phase, followed by concentric filling of the enhancement signal, and slow central enhancement of the signal in the delayed phase (12). Although CT and MR play an important role in the diagnosis of ICC, they have limitations in distant metastases, especially in soft tissue and occult metastases (13). Because some of the metastatic tumors often appear as isodense or slightly low-density nodules or masses in CT scans, it is easy to miss the diagnosis, and the CT and MRI manifestations of soft tissue metastatic tumors lack specificity, which makes it difficult to differentiate them from primary soft tissue malignant tumors. In this case, the clinical manifestation of the patient was right upper abdominal pain, and the serum tumor markers CA199 and CE125 were elevated, while other serological tests were normal, and the enhanced CT intrahepatic localization was consistent with the imaging characteristics of ICC. Enhanced CT intrahepatic localization was also consistent with the imaging features of ICC, but when evaluating metastasis, no obvious abnormality was found in intrahepatic and adjacent lymph nodes, and only multiple peritoneal nodules were detected, and no clear metastatic foci were detected in common distant metastatic sites, and only multiple peritoneal nodules were detected in the peritoneum and abdominal subcutaneous area with some circular enhancement, so PET-CT was performed to further assess the systemic metastasis. In addition to the abnormal increase in ^18^F-FDG uptake in the right lobe of the liver, peritoneal and abdominal subcutaneous nodes on enhanced CT, PET/CT also detected foci of increased metabolism of ^18^F-FDG in the left suprachoroidal femur, the right breast, thoracic subcutaneous area, the right upper arm, and the bilateral buttocks, of which the corresponding CTs of the right upper arm, bilateral buttocks, and left suprachoroidal femur did not show significant density changes. This case highlights the advantages of ^18^F-FDG PET/CT in comprehensively evaluating malignant metastases and detecting occult metastases, especially soft tissue metastases.

Lin et al. reported that the diagnostic value of PET/CT was superior to conventional imaging for N and M stages in patients with ICC. PET/CT plays an important role in different TNM staging by detecting new lesions, reassessing suspicious lesions, modifying tumor staging, and optimizing treatment strategies (14). According to the guidelines of the European Association for the Study of the Liver (EASL) and the American Association for the Study of Liver Diseases (AASLD), PET-CT is not suitable for primary staging because of its low accuracy. However, PET/CT has an important role in detecting lymph node metastasis and distant metastasis of ICC compared with CT and MRI and should be routinely performed in all patients with resectable disease (15, 16). PET/CT is a scan that combines anatomical imaging with metabolic visualization and readily reveals soft tissue metastases, especially before changes in soft tissue density and morphology, which are often difficult to visualize on CT scans, when PET reveals hypermetabolic nodules in soft tissue (17). As shown in this case, CT of the peritoneum, the left femur, the right upper arm, and the bilateral buttock muscles did not find obvious abnormalities, while PET showed obvious hypermetabolic nodules. Also, as a whole-body imaging modality, PET/CT scans include most of the musculoskeletal tissues of the body (usually from the base of the skull to the middle of the thighs, and sometimes from the top of the skull to the toes), whereas CT usually images only a limited area of the body (e.g., CT scans of the chest or neck only). PET has advantages over morphologic imaging techniques such as CT in providing a broader picture of the presence of metastases. Soft-tissue metastases generally appear in the later stages of the disease, and therefore the detection of soft-tissue metastases does not appear to have a significant impact on the staging of the cancer. However, in some cases, PET/CT imaging may be able to detect isolated, undetected skeletal muscle metastases, which may improve staging and affect treatment. PET/CT can provide the anatomical structure and location of tumors as well as metabolically active lesions. Distant metastatic or invasive tumors have been reported to be more malignant than primary tumors, and higher metabolic activity (higher SUV values) indicates a higher pathologic grade of the tumor. Guided by the SUV value of PET/CT, the efficiency of puncture can be improved, greater trauma can be avoided, and soft tissue metastasis may be the only feature in patients with tumors that lack typical presentation (13). PET/CT can stage cancer while searching for the primary focus, and it is a highly efficient, noninvasive, and highly sensitive test.

Optimal treatment requires accurate diagnosis, and pathologic findings remain the gold standard for ICC diagnosis. Puncture biopsy is undoubtedly the most effective method of obtaining pathologic tissue; tissue biopsy by ultrasound or CT-guided puncture is preferred. It is important to note that genetic testing is an essential complement to the pathologic diagnosis of ICC and contributes to drug selection for targeted therapy in ICC (11, 18). The results of histopathological and immunohistochemical examinations in our patient were diagnosed as poorly differentiated adenocarcinoma with CK7 and CK20 positivity. Radical surgical resection is currently the only way to cure ICC potentially, but due to its locally aggressive nature, this tumor is highly lethal, even with curative resection. Approximately 1/3 of patients with hepatobiliary malignancies have occult metastases or locally advanced review status (19, 20). Therefore, it is essential to determine tumor resectability by accurately staging tumor extent and metastases. Before surgical resection, a PET scan may be considered to help rule out occult primary disease and exclude another occult metastatic disease. In this disease, the presence of a metastatic soft tissue mass portends an advanced stage and poor prognosis. There are no specific guidelines for soft tissue metastases, and treatment regimens, including radiation, chemotherapy, surgical resection, and combination therapies, help relieve pain and reduce the size of metastatic lesions but have not yet improved the long-term prognosis of patients. For patients with advanced ICC, chemotherapeutic agents, mainly in combination with targeted and immunotherapy, are often the only option to improve prognosis and prolong survival. The chemotherapy regimen of gemcitabine plus cisplatin (GC) has been identified as the first-line standard chemotherapy regimen for cholangiocarcinoma, including ICC. It is the cornerstone of most current systemic treatment regimens (21).

In conclusion, ICC presenting with subcutaneous and skeletal muscle and breast metastases is very rare and has nonspecific clinical and radiologic features. Early diagnosis of soft tissue metastases is difficult, and metabolic imaging with ^18^F-FDG PET/CT helps to accurately identify the true metastases and their locations in soft tissues, which has great application value for staging tumors, determining treatment options, judging patients’ prognosis, and accurate pathology sampling. When progressive cutaneous or subcutaneous soft tissue lesions are encountered, clinicians should suspect visceral malignancy, examine them thoroughly, and consider ^18^F-FDG PET/CT for further evaluation. Also for these rare site lesions, in addition to considering the primary tumor, be aware of the possibility of distant metastases.

## Data Availability

The original contributions presented in the study are included in the article/supplementary material. Further inquiries can be directed to the corresponding author.

## References

[1] Banales JM Marin JJG Lamarca A Rodrigues PM Khan SA Roberts LR. Cholangiocarcinoma 2020: the next horizon in mechanisms and management. Nat Rev Gastroenterol Hepatol. (2020) 17:557–88. doi: 10.1038/s41575-020-0310-z PMC7447603 32606456

[2] Hyun SY Lee JH Shin HS Lee SW Park YN Park JY. Cutaneous metastasis from cholangiocarcinoma as the first clinical sign: A report of two cases. Gut Liver. (2011) 5:100–4. doi: 10.5009/gnl.2011.5.1.100 PMC3065084 21461082

[3] Tsurusaki M Kozuki R Urase A. (18)F-fluorodeoxyglucose positron emission tomography/computed tomography in intrahepatic cholangiocarcinoma: could it be a new paradigm? Hepatobiliary Surg Nutr. (2024) 13:379–81. doi: 10.21037/hbsn-23-675 PMC11007338 38617483

[4] Zizzo M Zanelli M Sanguedolce F Versari A Pattacini P Morini A. (18)F-fluorodeoxyglucose positron emission tomography for intrahepatic cholangiocarcinoma N- and M-staging: should guidelines recommend it at last? Hepatobiliary Surg Nutr. (2022) 11:789–92. doi: 10.21037/hbsn-22-273 PMC9577986 36268245

[5] Lee J Lee SW Han SY Baek YH Kim SY Rhyou HI. Rapidly aggravated skeletal muscle metastases from an intrahepatic cholangiocarcinoma. World J Gastroenterol. (2015) 21:1989–93. doi: 10.3748/wjg.v21.i6.1989 PMC4323479 25684968

[6] Kwon OS Jun DW Kim SH Chung MY Kim NI Song MH. Distant skeletal muscle metastasis from intrahepatic cholangiocarcinoma presenting as Budd-Chiari syndrome. World J Gastroenterol. (2007) 13:3141–3. doi: 10.3748/wjg.v13.i22.3141 PMC4172626 17589935

[7] Sukumar V Patkar S Goel M Sahay A. Intrahepatic cholangiocarcinoma presenting as a scalp mass. J Gastrointest Cancer. (2020) 51:1044–6. doi: 10.1007/s12029-020-00388-x 32152822

[8] Dogan G Karincaoglu Y Karincaoglu M Aydin NE. Scalp ulcer as first sign of cholangiocarcinoma. Am J Clin Dermatol. (2006) 7:387–9. doi: 10.2165/00128071-200607060-00008 17173474

[9] Esnaola NF Meyer JE Karachristos A Maranki JL Camp ER Denlinger CS. Evaluation and management of intrahepatic and extrahepatic cholangiocarcinoma. Cancer. (2016) 122:1349–69. doi: 10.1002/cncr.29692 26799932

[10] Zhang X Cai Y Xiong X Liu A Zhou R You Z. Comparison of current guidelines and consensus on the management of patients with cholangiocarcinoma: 2022 update. Intractable Rare Dis Res. (2022) 11:161–72. doi: 10.5582/irdr.2022.01109 PMC9709616 36457589

[11] Vogel A Bridgewater J Edeline J Kelley RK Klumpen HJ Malka D. Biliary tract cancer: Esmo clinical practice guideline for diagnosis, treatment and follow-up. Ann Oncol. (2023) 34:127–40. doi: 10.1016/j.annonc.2022.10.506 36372281

[12] Fabrega-Foster K Ghasabeh MA Pawlik TM Kamel IR. Multimodality imaging of intrahepatic cholangiocarcinoma. Hepatobiliary Surg Nutr. (2017) 6:67–78. doi: 10.21037/hbsn.2016.12.10 28503554 PMC5411278

[13] Ringe KI Wacker F. Radiological diagnosis in cholangiocarcinoma: application of computed tomography, magnetic resonance imaging, and positron emission tomography. Best Pract Res Clin Gastroenterol. (2015) 29:253–65. doi: 10.1016/j.bpg.2015.02.004 25966426

[14] Lin Y Chong H Song G Zhang C Dong L Aye L. The influence of (18)F-fluorodeoxyglucose positron emission tomography/computed tomography on the N- and M-staging and subsequent clinical management of intrahepatic cholangiocarcinoma. Hepatobiliary Surg Nutr. (2022) 11:684–95. doi: 10.21037/hbsn-21-25 PMC9577996 36268256

[15] Bowlus CL Arrive L Bergquist A Deneau M Forman L Ilyas SI. Aasld practice guidance on primary sclerosing cholangitis and cholangiocarcinoma. Hepatology. (2023) 77:659–702. doi: 10.1002/hep.32771 36083140

[16] European Association for the Study of the Liver. Electronic address eee, European Association for the Study of the L. Easl-Ilca Clinical Practice Guidelines on the Management of Intrahepatic Cholangiocarcinoma. J Hepatol. (2023) 79:181–208. doi: 10.1016/j.jhep.2023.03.010 37084797

[17] Qiu DS Xu LY Shames S. The value of (18)F-fluorodeoxyglucose positron emission tomography combined with computed tomography in the detection and characterization of soft tissue metastasis. Mol Clin Oncol. (2014) 2:761–6. doi: 10.3892/mco.2014.312 PMC4106726 25054043

[18] Moris D Palta M Kim C Allen PJ Morse MA Lidsky ME. Advances in the treatment of intrahepatic cholangiocarcinoma: an overview of the current and future therapeutic landscape for clinicians. CA Cancer J Clin. (2023) 73:198–222. doi: 10.3322/caac.21759 36260350

[19] D’Angelica M Fong Y Weber S Gonen M DeMatteo RP Conlon K. The role of staging laparoscopy in hepatobiliary Malignancy: prospective analysis of 401 cases. Ann Surg Oncol. (2003) 10:183–9. doi: 10.1245/aso.2003.03.091 12620915

[20] Goere D Wagholikar GD Pessaux P Carrere N Sibert A Vilgrain V. Utility of staging laparoscopy in subsets of biliary cancers: laparoscopy is a powerful diagnostic tool in patients with intrahepatic and gallbladder carcinoma. Surg Endosc. (2006) 20:721–5. doi: 10.1007/s00464-005-0583-x 16508808

[21] Qu WF Liu WR Shi YH. Adjuvant chemotherapy for intrahepatic cholangiocarcinoma: far from a clinical consensus. Hepatobiliary Surg Nutr. (2021) 10:887–9. doi: 10.21037/hbsn-21-362 PMC8683926 35004963

